# Angle Estimation of Simultaneous Orthogonal Rotations from 3D Gyroscope Measurements

**DOI:** 10.3390/s110908536

**Published:** 2011-09-02

**Authors:** Sara Stančin, Sašo Tomažič

**Affiliations:** Faculty of Electrical Engineering, University of Ljubljana, Ljubljana 1000, Slovenia; E-Mail: saso.tomazic@fe.uni-lj.si

**Keywords:** gyroscope, angular velocity, angular orientation, simultaneous rotations, rotation axis, rotation angle, spatial angle

## Abstract

A 3D gyroscope provides measurements of angular velocities around its three intrinsic orthogonal axes, enabling angular orientation estimation. Because the measured angular velocities represent simultaneous rotations, it is not appropriate to consider them sequentially. Rotations in general are not commutative, and each possible rotation sequence has a different resulting angular orientation. None of these angular orientations is the correct simultaneous rotation result. However, every angular orientation can be represented by a single rotation. This paper presents an analytic derivation of the axis and angle of the single rotation equivalent to three simultaneous rotations around orthogonal axes when the measured angular velocities or their proportions are approximately constant. Based on the resulting expressions, a vector called the simultaneous orthogonal rotations angle (SORA) is defined, with components equal to the angles of three simultaneous rotations around coordinate system axes. The orientation and magnitude of this vector are equal to the equivalent single rotation axis and angle, respectively. As long as the orientation of the actual rotation axis is constant, given the SORA, the angular orientation of a rigid body can be calculated in a single step, thus making it possible to avoid computing the iterative infinitesimal rotation approximation. The performed test measurements confirm the validity of the SORA concept. SORA is simple and well-suited for use in the real-time calculation of angular orientation based on angular velocity measurements derived using a gyroscope. Moreover, because of its demonstrated simplicity, SORA can also be used in general angular orientation notation.

## 1. Introduction

Gyroscopes provide angular velocity measurements with respect to inertial space. With recent developments in gyroscope technology, their usage in various fields is observably increasing. For example, highly accurate fibre optic gyroscopes [1] are used in different aerospace and missile navigation applications. These gyroscopes use the interference of light to measure angular velocity. Microelectromechanical system (MEMS) gyroscopes [2,3] use vibrating mechanical elements to sense rotation and have no rotating parts. The operating principle governing the use of MEMS gyroscopes is the Coriolis effect. MEMS sensors have several advantages: their small size, light weight, low power consumption, low costs, and ease of design and use. Such gyroscopes can be used, for example, in automobiles (for ride stabilisation and rollover detection [4]), robotics (in state estimation for legged robots [5]), biomedical applications (for motion capture and motion pattern classification and characterisation; e.g., [6–10]), and virtual and augmented reality applications (e.g., [11,12]). Owing to their low battery consumption and small size, MEMS sensors are a promising tool for outdoor measurements and ambulatory monitoring [6]. Because of their low cost, they are suitable for a wide range of commercial applications [2].

In combination with accelerometers, gyroscopes are used in position, velocity, and attitude computation in a variety of navigation and motion tracking applications for aircraft, land and underwater vehicles, and robots (e.g., [13–17]) and in human motion tracking (e.g., [6,18]).

**Figure 1.**
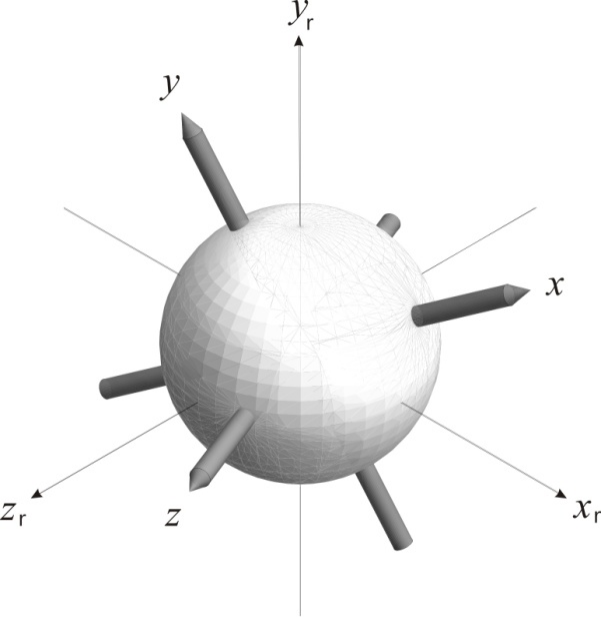
Reference and 3D gyroscope intrinsic coordinate systems, denoted by *x*_r_, *y*_r_, *z*_r_ and *x*, *y*, *z*, respectively.

By providing angular velocity measurements, gyroscopes can also be used in angular orientation estimation. In general, angular orientation is the position of a rigid body intrinsic coordinate system relative to a reference coordinate system with the same origin. It can be indicated with a rotation needed to move the first system, initially aligned with the second, to its new position. The reference and intrinsic coordinate systems are considered Cartesian, with axes denoted with *x*_r_, *y*_r_, *z*_r_ and *x*, *y*, *z*, respectively, as illustrated in Figure 1. The orientation of the coordinate system axes conforms to the right-hand rule.

In gyroscope measurement, the gyroscope is considered the rigid body and inertial space the reference system; the measured angular velocities indicate the rotation needed to move the sensor to its new position. To fully specify angular orientation in inertial space, the gyroscope must be suitable for measuring angular velocities around all three intrinsic coordinate system axes (*i.e.*, it must be a 3D gyroscope). For the coordinate systems described and illustrated in Figure 1, 3D gyroscope angular velocity outputs will represent simultaneous rotations around axes of sensitivity *x*, *y* and *z*.

Because rotations in general are not commutative, one cannot treat the three simultaneous angular velocities measured with a 3D gyroscope as sequential rotations. There are six possible sequences of three orthogonal rotations and, thus, six different angular orientations. None of them are correct. Infinitesimally small rotations have been shown to be commutative [19]. Thus, when the angles of the three simultaneous rotations are small, the difference between the six abovementioned angular orientations became negligible because the rotations become nearly commutative.

Therefore, the effect of the three simultaneous rotations can be approximated by a sequence of sufficiently small sequential rotations around orthogonal axes. Which of the six possible sequences is used for individual rotations is of no importance. The smaller the angles of the individual rotations, the smaller the estimated angular orientation error. However, more processing power and time are required.

The three simultaneous orthogonal rotations measured with a 3D gyroscope represent a single rotation around a certain axis for a certain angle *φ*. This rotation and the resulting (spatial) angular orientation can be uniquely represented using a rotation vector, *i.e.*, the vector aligned with the rotation axis and with the length equal to the rotation angle *φ*. In this paper, we show that when the measured angular velocities or their proportions are constant, the components of this vector are the angles of the three simultaneous orthogonal rotations. For this reason, we call this vector the simultaneous orthogonal rotations angle (SORA). We first introduced SORA in [20]; there, however, we only presented numerical verification that SORA was the rotation vector of the equivalent single rotation. In this paper, these results are derived. This derivation is achieved using infinitesimally small rotations.

Based on SORA, the angular orientation of a rigid body can be calculated from the measured angular velocities when they or their proportions are approximately constant. This process requires just one step and thus avoids computing the infinitesimal rotation approximation.

SORA indicates a unique angular orientation of an object of interest in inertial space. Because of its simplicity, not only is it suitable for the real-time calculation of angular orientation based on angular velocity measurements obtained using a gyroscope, but it is also useful for general angular orientation notation.

This paper is organised as follows. In Section 2, we present the derivation of the axis and angle of the single rotation equivalent to the three simultaneous rotations around orthogonal axes. On this basis, we define SORA and emphasise its applicability. For clarity of presentation, all longer derivations are presented in the appendix. In Section 3, we describe the test measurements performed to validate the SORA concept and examine the accuracy of the angular orientation estimates obtained using SORA. In Section 4, we draw our conclusions.

## 2. Simultaneous Orthogonal Rotations Angle

Let us consider a 3D gyroscope providing measurements of angular velocities *ω*_x_, *ω*_y_ and *ω*_z_ around its three intrinsic orthogonal axes, *x*, *y* and *z*, respectively. Suppose now that all three angular velocities are constant during some time interval T, and that the axes of the gyroscope intrinsic coordinate system are aligned with the axes of the reference coordinate system at the beginning of this interval.

Any angular orientation can be represented using a single rotation around a certain axis that rotates the object from its initial position to its new position. For this reason, the angular orientation of the gyroscope at the end of interval *T* can be represented using a rotation matrix **R**(*φ*, **v**), where *φ* is the rotation angle in the positive direction around the rotation axis defined by the unit vector **v**. For the definition of a rotation matrix, see, for example [21].

If we observe the rotating gyroscope in the reference coordinate system, we will see that the orientations of the rotation axes constantly change, which makes further analysis more difficult. Thus, it is much more convenient to examine the rotation of the reference frame in the gyroscope intrinsic coordinate system. In this case, the orientations of the rotation axes do not change. The reference frame rotates around them with the same angular velocities in the opposite direction, *i.e.*, −*ω_x_*, −*ω_y_*, and −*ω_z_*. Because both coordinate systems are initially aligned, the rotation axis of the equivalent rotation is the same regardless of which coordinate system we are observing. However, the rotation angle of the equivalent rotation is now equal to −*φ*.

We have shown that simultaneous rotations of the reference coordinate system with angular velocities −*ω_x_*, −*ω_y_*, and −*ω_z_* are equivalent to a rotation of angle −*φ* around vector **v**. It is thus obvious that simultaneous rotations of the reference coordinate system with angular velocities *ω_x_*, *ω_y_*, and *ω_z_* are equivalent to a rotation of angle *φ* around vector **v** and that they can be described using the same rotation matrix **R**(*φ*, **v**) as the rotation of the gyroscope in the reference coordinate system. In the following paragraphs, we will determine *φ* and **v** which is our aim because *φ* and **v** uniquely define the angular orientation of the sensor at the end of the observation interval *T*.

Suppose now that the reference coordinate system is rotating simultaneously around sensor intrinsic axes *x, y,* and *z* with angular velocities *ω_x_*, *ω_y_*, and *ω_z_*. During this interval, the reference frame turns for angles

(1) $$\varphi_{x}=\omega_{x}T;\varphi_{y}=\omega_{y}T;\varphi_{z}=\omega_{z}T$$

Due to rotation non-commutativity, **R**(*φ*, **v**) cannot be obtained by considering these three simultaneous rotations sequentially. Therefore, we must decompose the total rotation into a sequence of *n* small rotations of duration *T/n*. During each small rotation, the reference coordinate system simultaneously turns around the gyroscope intrinsic axes *x*, *y*, and *z* for angles *φ_x_*/*n*, *φ_y_*/*n*, and *φ_z_*/*n*, respectively. Because all of these small rotations are equal, the axis and angle of their equivalent single rotation are the same. Furthermore, because angular velocities *ω_x_*, *ω_y_*, and *ω_z_* now refer to the reference frame rotations and because the orientations of the rotation axes do not change, the axis of this equivalent rotation again coincides with vector **v**. During *T/n*, the reference frame rotates around this axis for angle *φ*/*n*, so we denote the corresponding rotation matrix as **R**(*φ*/*n*, **v**). It then holds that:

(2) $$R(\varphi ,v)=R^{n}(\varphi /n,v)$$

If *n* is sufficiently large, such that rotations become nearly commutative, **R**(*φ*/*n*, **v**) can be approximated based on simultaneous rotations for angles *φ_x_*/*n*, *φ_y_*/*n*, and *φ_z_*/*n*, sequentially and in any preferred order. Thus, we can write:

(3) $$R(\Delta \varphi_{\text{app}}(n),v_{\text{app}}(n))=R(\varphi_{x}/n,u_{x})\cdot R(\varphi_{y}/n,\;u_{y})\cdot R(\varphi_{z}/n,u_{z})$$

where **v**_app_(*n*) and Δ*φ*_app_(*n*) denote approximations of **v** and *φ*/*n*, respectively, and **u***_x_*, **u***_y_*, and **u***_z_* are the base vectors of the gyroscope intrinsic coordinate system.

Let us now increase *n* beyond all limits so that small rotations become infinitesimally small and thus commutative [19]. The above approximations **v**_app_(*n*) and Δ*φ*_app_(*n*) then approach exact values of **v** and *φ*/*n*. It holds that:

(4) $$v=\underset{n\to \infty }{\text{lim}}\left(v_{\text{app}}(n)\right)=\frac{1}{\sqrt{\varphi_{x}^{2}+\varphi_{y}^{2}+\varphi_{z}^{2}}}\left[\begin{array}{c} \varphi_{x}\\ \varphi_{y}\\ \varphi_{z} \end{array}\right]$$

and

(5) $$\varphi =\underset{n\to \infty }{\text{lim}}\left(n\cdot \Delta \varphi_{\text{app}}(n)\right)=\sqrt{\varphi_{x}^{2}+\varphi_{y}^{2}+\varphi_{z}^{2}}$$

For the derivation of the above results, see Appendix A.

This step completes the equivalent rotation axis and angle derivation. From Equations (4) and (5), we can obtain the rotation vector Φ, *i.e.*, the vector aligned with the rotation axis with a length that is equal to the rotation angle:

(6) $$\Phi =\varphi \cdot v=\left[\begin{array}{c} \varphi_{x}\\ \varphi_{y}\\ \varphi_{z} \end{array}\right]$$

Because we derived this vector from simultaneous rotations around orthogonal axes, we named it the simultaneous orthogonal rotations angle (SORA). The components of SORA are the angles of three simultaneous rotations around coordinate system axes. The orientation and magnitude of this vector are equal to the equivalent single rotation axis and angle, respectively. As long as our initial condition that angular velocities must be constant is met, SORA will represent the resulting angular orientation of three simultaneous rotations around orthogonal axes.

In fact, the previously stated holds as long as the orientation of the rotation axis is constant. This is true even if the measured angular velocities change, as long as their proportions are the same. Then, in each of the *n* small rotation steps, we can choose such a *T/n*, that all three rotation angles *φ_x_*/*n*, *φ_y_*/*n*, and *φ_z_*/*n* stay the same. However, if the measured angular velocities do not change proportionally, the orientation of the rotation axis also changes.

SORA indicates a unique angular orientation of an object of interest relative to some reference frame. Because of its simplicity, it is both suitable for the real-time calculation of angular orientation based on angular velocity measurements obtained using a gyroscope and useful for general angular orientation notation.

Using gyroscope measurements that provide rotation angle estimation, it is also reasonable to combine angular velocities around three orthogonal axes in vector form:

(7) $$\Omega =\frac{\Phi }{T}=\left[\begin{array}{c} \omega_{x}\\ \omega_{y}\\ \omega_{z} \end{array}\right]$$

Such notation is appropriate for a 3D gyroscope sensor because the components of the angular velocity vector Ω in Equation (7) are its outputs. When the measured angular velocities or their proportions are approximately constant, the orientation of the rotation axis is also constant. Then, the axis of the equivalent single rotation is equal to the axis of actual gyroscope rotation. For this reason, Ω is equal to gyroscope angular velocity represented in vector form. It is orthogonal to the rotation plane, and its magnitude is equal to the angular velocity in that plane.

## 3. Test Measurements

**Figure 2.**
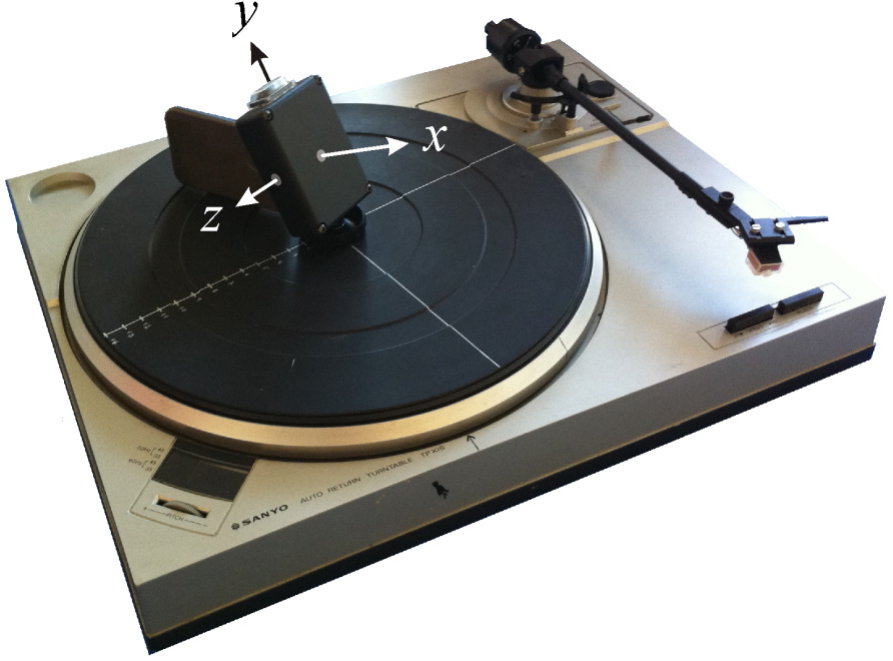
Vector SORA proof-of-concept measurement scheme. The reference coordinate system axes *x*_r_ *y*_r_, and *z*_r_ are initially aligned with the intrinsic axes *x y* and *z.*

To confirm the validity of the SORA concept, we performed test measurements using a stable rotation speed gramophone and a 3D gyroscope and accelerometer sensor mounted on the gramophone rotation disk. The test measurement scheme and the intrinsic *x y z* and reference *x*_r_ *y*_r_ *z*_r_ coordinate systems are illustrated in Figure 2.

We used a Sanyo turntable gramophone and set the turntable rotation speed to 45 r.p.m. The 3D sensor was comprised of one dual-axis ST LPR530AL gyroscope, one single-axis ST LPY530AL gyroscope and one three-axis ST LIS331HH linear accelerometer. The sensor was constructed in such a way that its sensitive axes were mutually orthogonal. Both of the MEMS gyroscopes use a sensing principle based on the measurement of the Coriolis forces. The gyroscopes convert angular velocity information into an analogue DC voltage at its output. The MEMS accelerometer output corresponding to each axis was an analogue voltage proportional to the projection of the total acceleration of the sensor along its direction. The analogue output of both gyroscopes and the accelerometer was sent to an analogue-to-digital converter. A wireless module was used to capture sensor digital outputs, which were stored on a laptop. The sampling rate was 1,024 Hz. The gyroscope and accelerometer outputs were obtained in units of [°/s] and [mg], respectively. According to the device specifications, the maximum measurable angular velocity is ±300°/s, whereas the accelerometer measurement range is ±6/±12/±24 g. Zero-level measurements were performed when there were no angular velocities or external forces present. Mean values obtained were subtracted from all further measurements.

We also performed sensor calibration. The following procedure was repeated for each sensor intrinsic axis. The sensor was oriented so that its sensitive axis was aligned with the gravity force. The ratio between the known gravity constant and the mean measurement output value when the sensor was at rest was used as the accelerometer scaling factor. To obtain the gyroscope scaling factor, the sensor was rotated at the centre of the gramophone rotation disk around that axis. The ratio between the known gramophone rotation disk speed and the mean measurement output value was used as the gyroscope scaling factor.

### 3.1. Test Measurement Results

**Figure 3.**
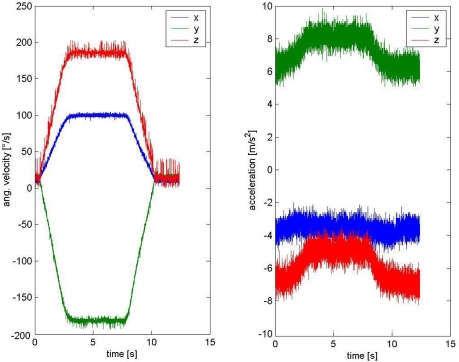
Sensor angular velocity and acceleration outputs during rotation around an arbitrary axis.

To validate the SORA concept, we performed rotation measurements for several arbitrary orientations of the sensor relative to the rotating disk at the disk centre. One such measurement output set is presented in Figure 3.

Gyroscope measurement outputs *ω_x_*, *ω_y_*, and *ω_z_* obtained during constant velocity rotation periods were used for SORA calculations according to Equation (6). During *T*_rot_ = 1 s, we obtained *N*_rot_ = 1,024 samples, which we averaged:

(8) $$\Phi =\left[\begin{array}{c} \varphi_{x}\\ \varphi_{y}\\ \varphi_{z} \end{array}\right]=\left[\begin{array}{c} \bar{\omega_{x}}\\ \bar{\omega_{y}}\\ \bar{\omega_{z}} \end{array}\right]\cdot T_{\text{rot}}=\frac{T_{\text{rot}}}{N_{\text{rot}}}\sum_{i=1}^{N_{\text{rot}}}\left[\begin{array}{c} \omega_{x}\\ \omega_{y}\\ \omega_{z} \end{array}\right]$$

Based on Equation (8), we calculated the equivalent rotation axis and angle of *T*_rot_ = 1 s rotation following Equations (4) and (5), respectively. We obtained the following results:

(9) $$v=\left[\begin{array}{c} 0.3233\\ -0.7145\\ 0.6205 \end{array}\right]$$

and:

(10) $$\varphi =270{}^{\circ}28^{\prime }8^{\prime \prime }$$

We then matched the results obtained to the actual sensor rotation axis and angle. For specific sensor orientation during the rest period, we used gravity projections on sensor intrinsic axes that were determined based on accelerometer measurement outputs *α_x_*, *α_y_*, and *α_z_* to determine the actual gramophone rotation axis orientation as expressed in the sensor intrinsic coordinate system. During *T*_rest_ = 1 s, we obtained *N*_rest_ = 1,024 samples, which we averaged:

(11) $$v_{\text{act}}=-\frac{\bar{a}}{\left\|\bar{a}\right\|};\;\bar{a}=\left[\begin{array}{c} \bar{a_{x}}\\ \bar{a_{y}}\\ \bar{a_{z}} \end{array}\right]=\frac{1}{N_{\text{rest}}}\sum_{i=1}^{N_{\text{rest}}}\left[\begin{array}{c} \bar{a_{x}}\\ \bar{a_{y}}\\ \bar{a_{z}} \end{array}\right]$$

The actual rotation angle was obtained based on the gramophone rotation disk angular velocity of 45 r.p.m.:

(12) $$\varphi_{\text{act}}=\omega_{\text{act}}\cdot T_{\text{rot}};\omega_{\text{act}}=4.7124s^{-1}$$

Using Equations (11) and (12), we obtained the following results:

(13) $$v_{\text{act}}=\left[\begin{array}{c} 0.3627\\ -0.7112\\ 0.6022 \end{array}\right]$$

and

(14) $$\varphi_{\text{act}}=270{}^{\circ}$$

The relative norm error of the rotation axis of Equation (9) is:

(15) $${\mathrm{err}}_{v}=\frac{\left\|v-v_{\text{act}}\right\|}{\left\|v_{\text{act}}\right\|}=\left\|\left[\begin{array}{c} -0.0394\\ 0.0033\\ 0.0183 \end{array}\right]\right\|=0.0436$$

while the relative rotation angle Equation (10) error is:

(16) $${\mathrm{err}}_{\varphi }=\frac{\varphi -\varphi_{\text{act}}}{\varphi_{\text{act}}}=0.0018$$

We performed 10 measurement instances. According to the Kolmogorov-Smirnov test at significance level α = 0.05, the null-hypothesis that random errors *Err_v_* and *Err_φ_* are normal, could not be rejected. For both errors we obtained the following statistics:

(17) $$\begin{array}{l} \text{mean}({\mathrm{Err}}_{v})=0.0753\\ \text{std}({\mathrm{Err}}_{v})=0.0422\\ \text{mean}({\mathrm{Err}}_{\varphi })=0.0056\\ \text{std}({\mathrm{Err}}_{\varphi })=0.0117 \end{array}$$

## 4. Conclusions

The concept of a vector simultaneous orthogonal rotations angle (SORA), as presented in this paper, is well suited for calculating angular orientation based on 3D gyroscope measurement outputs that are approximately constant or in constant proportion. When this condition is met, the orientation of the rotation axis does not change and the components of SORA are equal to the angles of three simultaneous rotations measured with a gyroscope. The three simultaneous rotations resulting from angular orientation can be represented with one single rotation. A derivation of this equivalent single rotation axis and angle is provided here. The axis of the equivalent single rotation is aligned with SORA, whereas the rotation angle is equal to its magnitude. Therefore, SORA is actually a rotation vector.

According to the newly proposed vector SORA, as long as angular velocities or their proportions are approximately constant, angular orientation calculation only requires a single step. This fact makes it possible to avoid computing the iterative infinitesimal rotation approximation. Test measurements performed show that SORA is suitable for angular orientation estimation when average values of approximately constant angular velocities are considered.

SORA is appropriate for use for a number of applications that require angular orientation information, including navigation and motion tracking. As has been confirmed using test measurements, SORA is simple and suitable for the real-time calculation of angular orientation based on gyroscope angular velocity measurements.

Not only is SORA well suited for representing angular orientation based on actual gyroscope measurements, but it can also be used in generalised angular orientation notation. In representing angular orientation with simultaneous rotations, we can avoid the problem of rotation non-commutativity. The three simultaneous rotation angles, as included in SORA, represent a unique angular orientation, while considering only right-hand rotations around three orthogonal axes yields six different rotation sequences. Each of these rotation sequences specifies a different angular orientation. Because of this, SORA is much more suitable for rotation presentation purpose than widely used Euler angles. Moreover, SORA helps interpret the meaning of the rotation vector with measurable quantities.

## Figures and Tables

**Figure A.1.**
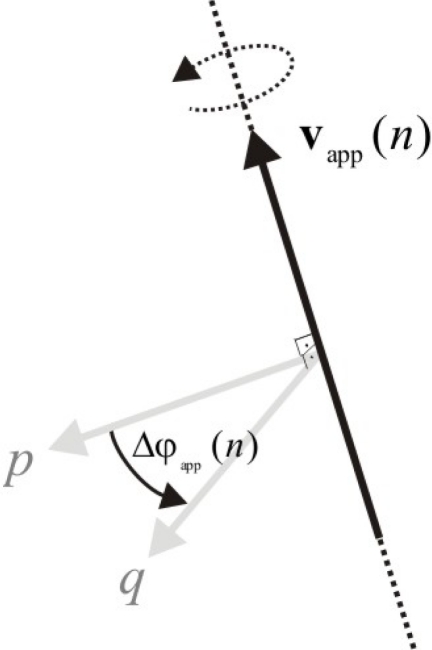
Rotation angle determination.

## Appendix

### Derivation of Equivalent Single Rotation Axis and Angle

Let us consider simultaneous rotations around three orthogonal axes *x*, *y*, and *z* for angles *φ_x_*, *φ_y_*, and *φ_z_*, and let us represent their composite using a rotation matrix **R**(*φ*, **v**), where *φ* is the rotation angle in the positive direction around the rotation axis defined by the unit vector **v**. Due to rotation non-commutativity, **R**(*φ*, **v**) cannot be obtained by considering these three simultaneous rotations sequentially. Therefore, we decompose the total rotation into a sequence of *n* small rotations in which each such small rotation includes rotations for angles *φ_x_*/*n*, *φ_y_*/*n*, and *φ_z_*/*n*. Because all of these small rotations are equal, the axis and angle of their equivalent single rotation are the same. It then holds that:

(A.1) $$R(\varphi ,v)=R^{n}(\varphi /n,v)$$

If *n* is sufficiently large that the rotations become nearly commutative, **R**(*φ*, **v**) can be approximated using simultaneous rotations for angles *φ_x_*/*n*, *φ_y_*/*n*, and *φ_z_*/*n*, sequentially and in any preferred order. Thus, we can write:

(A.2) $$R(\varphi ,v)=R^{n}(\Delta \varphi_{\text{app}}(n),v_{\text{app}}(n))$$

(A.3) $$R(\Delta \varphi_{\text{app}}(n),v_{\text{app}}(n))=R(\varphi_{x}/n,u_{x})\cdot R(\varphi_{y}/n,u_{y})\cdot R(\varphi_{z}/n,u_{z})$$

where **v**_app_(*n*) and Δ*φ*_app_(n) denote approximations of **v** and *φ*/*n*, respectively; **u***_x_*, **u***_y_*, and **u***_z_* denote the *x y z* coordinate system base vector; and **R**(*φ_x_*/*n*, **u***_x_*), **R**(*φ_y_*/*n*, **u***_y_*), **R**(*φ_z_*/*n*, **u***_z_*), represent rotation matrices:

(A.4) $$R(\varphi_{x}/n,u_{x})=\left[\begin{array}{ccc} 1 & 0 & 0\\ 0 & \text{cos}(\varphi_{x}/n) & -\text{sin}(\varphi_{x}/n)\\ 0 & \text{sin}(\varphi_{x}/n) & \text{cos}(\varphi_{x}/n) \end{array}\right]$$

(A.5) $$R(\varphi_{y}/n,u_{y})=\left[\begin{array}{ccc} \text{cos}(\varphi_{y}/n) & 0 & \text{sin}(\varphi_{y}/n)\\ 0 & 1 & 0\\ -\text{sin}(\varphi_{y}/n) & 0 & \text{cos}(\varphi_{y}/n) \end{array}\right]$$

(A.6) $$R(\varphi_{z}/n,u_{z})=\left[\begin{array}{ccc} \text{cos}(\varphi_{z}/n) & -\text{sin}(\varphi_{z}/n) & 0\\ \text{sin}(\varphi_{z}/n) & \text{cos}(\varphi_{z}/n) & 0\\ 0 & 0 & 1 \end{array}\right]$$

Inserting Equations (A.4)–(A.6) into Equation (A.4) yields

(A.7) $$\begin{array}{l} R(\Delta \varphi_{\text{app}}(n),v_{\text{app}}(n))=R(\varphi_{x}/n,u_{x})\cdot R(\varphi_{y}/n,u_{y})\cdot R(\varphi_{z}/n,u_{z})=\\ \left[\begin{array}{ccc} c\left(\frac{\varphi_{y}}{n}\right)\cdot c\left(\frac{\varphi_{z}}{n}\right) & -c\left(\frac{\varphi_{y}}{n}\right)\cdot s\left(\frac{\varphi_{z}}{n}\right) & s\left(\frac{\varphi_{y}}{n}\right)\\ s\left(\frac{\varphi_{x}}{n}\right)\cdot s\left(\frac{\varphi_{y}}{n}\right)\cdot c\left(\frac{\varphi_{z}}{n}\right)+c\left(\frac{\varphi_{x}}{n}\right)\cdot s\left(\frac{\varphi_{z}}{n}\right) & c\left(\frac{\varphi_{x}}{n}\right)\cdot c\left(\frac{\varphi_{z}}{n}\right)-s\left(\frac{\varphi_{x}}{n}\right)\cdot s\left(\frac{\varphi_{y}}{n}\right)\cdot s\left(\frac{\varphi_{z}}{n}\right) & -s\left(\frac{\varphi_{x}}{n}\right)\cdot c\left(\frac{\varphi_{y}}{n}\right)\\ -c\left(\frac{\varphi_{x}}{n}\right)\cdot s\left(\frac{\varphi_{y}}{n}\right)\cdot c\left(\frac{\varphi_{z}}{n}\right)+s\left(\frac{\varphi_{x}}{n}\right)\cdot s\left(\frac{\varphi_{z}}{n}\right) & s\left(\frac{\varphi_{x}}{n}\right)\cdot c\left(\frac{\varphi_{z}}{n}\right)+c\left(\frac{\varphi_{x}}{n}\right)\cdot s\left(\frac{\varphi_{y}}{n}\right)\cdot s\left(\frac{\varphi_{z}}{n}\right) & c\left(\frac{\varphi_{x}}{n}\right)\cdot c\left(\frac{\varphi_{y}}{n}\right) \end{array}\right] \end{array}$$

where *c* and *s* represent the cosine and sine trigonometric functions according to:

(A.8) $$\begin{array}{l} c(\alpha )=\text{cos}(\alpha )\\ s(\alpha )=\text{sin}(\alpha ) \end{array}$$

Rotating a vector **v**_app_(*n*) coinciding with the rotation axis results in **v**_app_(*n*) itself. Thus, we can write:

(A.9) $$R(\Delta \varphi_{\text{app}}(n),v_{\text{app}}(n))\cdot v_{\text{app}}(n)=v_{\text{app}}(n)$$

Based on Equation (A.9), it is obvious that vector **v**_app_(*n*) is the rotation matrix **R**(Δ*φ*_app_(n), **v**_app_(*n*)) eigenvector for the eigenvalue λ = 1 (every rotation matrix has such an eigenvalue [19]):

(A.10) $$(R(\Delta \varphi_{\text{app}}(n),v_{\text{app}}(n))-I)\cdot v_{\text{app}}(n)=0$$

To obtain the rotation angle Δ*φ*_app_(n) from **R**(Δ*φ*_app_(n), **v**_app_(*n*)), let us choose an arbitrary vector **p** orthogonal to **v** and perform an **R**(Δ*φ*_app_(n), **v**_app_(*n*)) rotation of this vector:

(A.11) $$q=R(\Delta \varphi_{\text{app}}(n),v_{\text{app}}(n))\cdot p$$

where **q** is the newly obtained vector as illustrated in Figure A.1. Because **p** and **q** are both orthogonal to the rotation axis, the angle between them is equal to the rotation angle *φ*/*n*. If these vectors are normalised, we can write:

(A.12) $$\text{cos}(\Delta \varphi_{\text{app}}(n))=p\cdot q$$

(A.13) $$\begin{array}{l} \left\|p\right\|=1\\ \left\|q\right\|=1 \end{array}$$

Increasing n beyond all limits, we find that the small rotations become infinitesimally small and thus commutative [20]. The approximations of **v**_app_(*n*) and *φ*_app_(n) then approach the exact values for **v** and *φ*/*n*:

(A.14) $$v=\underset{n\to \infty }{\text{lim}}(v_{\text{app}}(n))$$

(A.15) $$\varphi =\underset{n\to \infty }{\text{lim}}(n\cdot \Delta \varphi_{\text{app}}(n))$$

Given Equation (A.10) and Equation (A.14), we can then write:

(A.16) $$(\underset{n\to \infty }{\text{lim}}(R(\Delta \varphi_{\text{app}}(n),v_{\text{app}}(n)))-I)\cdot v=0$$

Inserting Equation (A.7) into the above eigenvector system of equations and solving it for a normalised vector **v** using Mathematica [22] yields:

(A.17) $$v=\frac{1}{\sqrt{\varphi_{x}^{2}+\varphi_{y}^{2}+\varphi_{z}^{2}}}\left[\begin{array}{c} \varphi_{x}\\ \varphi_{y}\\ \varphi_{z} \end{array}\right]$$

From Equation (A.12) and Equation (A.15), it follows that:

(A.18) $$\varphi =\underset{n\to \infty }{\text{lim}}(n\cdot {\text{cos}}^{-1}(p\cdot q))$$

Using the l’Hospital rule for limits, the above equation can be expressed as:

(A.19) $$\varphi =\underset{n\to \infty }{\text{lim}}\left(\frac{\frac{d}{dn}({\text{cos}}^{-1}(p\cdot q))}{\frac{d}{\mathrm{dn}}(\frac{1}{n})}\right)=\underset{n\to \infty }{\text{lim}}\left(\frac{-\frac{1}{\sqrt{1-{\left(p\cdot q\right)}^{2}}}\cdot \frac{d}{\mathrm{dn}}(p\cdot q)}{-\frac{1}{n^{2}}}\right)$$

A vector orthogonal to **v**_app_(*n*) is also orthogonal to **v** in limit Equation (A.14). We can then set:

(A.20) $$p=v⊗\left[\begin{array}{c} 1\\ 0\\ 0 \end{array}\right];p=\frac{p}{\left\|p\right\|}$$

where **v** is defined according to Equation (A.17). It should be noted that we are considering general simultaneous rotations around all three coordinate system axes. Where *φ_y_* = 0 and *φ_z_* = 0, the above equation must be suitably changed to obtain the required vector orthogonality, for example:

$$p=v⊗\left[\begin{array}{c} 0\\ 1\\ 0 \end{array}\right]$$

Inserting normalised vectors **p** and **q** given with Equations (A.20), (A.17), and (A.11) into Equation (A.19) yields the following expression:

(A.21) $$\varphi =\underset{n\to \infty }{\text{lim}}\frac{A+B+C+D+E}{\sqrt{F^{2}-{\left(G+H-I\right)}^{2}}}$$

where:

(A.22) $$\begin{array}{l} A=\text{cos}\frac{\varphi_{x}}{n}\cdot \left(\varphi_{y}^{3}\cdot \text{sin}\frac{\varphi_{y}}{n}+\varphi_{y}\cdot \varphi_{z}\cdot \text{cos}\frac{\varphi_{z}}{n}\cdot (\varphi_{x}+\varphi_{z}\cdot \text{sin}\frac{\varphi_{y}}{n})\right)\\ B=\varphi_{z}^{2}\cdot \text{cos}\frac{\varphi_{x}}{n}\cdot \text{sin}\frac{\varphi_{z}}{n}\cdot (\varphi_{z}+\varphi_{z}\cdot \text{sin}\frac{\varphi_{y}}{n})\\ C=\varphi_{y}\cdot \varphi_{z}\cdot \text{cos}\frac{\varphi_{x}}{n}\cdot \text{cos}\frac{\varphi_{y}}{n}\cdot (-\varphi_{x}+\varphi_{y}\cdot \text{sin}\frac{\varphi_{z}}{n})\\ D=\varphi_{y}\cdot \text{sin}\frac{\varphi_{x}}{n}\cdot \text{cos}\frac{\varphi_{y}}{n}\cdot (\varphi_{x}\cdot \varphi_{y}+\varphi_{z}^{2}\cdot \text{sin}\frac{\varphi_{z}}{n})\\ E=\varphi_{z}\cdot \text{sin}\frac{\varphi_{x}}{n}\cdot \left(\varphi_{y}^{2}\cdot \text{sin}\frac{\varphi_{y}}{n}+\varphi_{z}\cdot \text{cos}\frac{\varphi_{z}}{n}\cdot (\varphi_{x}+\varphi_{z}\cdot \text{sin}\frac{\varphi_{y}}{n})-\varphi_{y}\cdot \text{sin}\frac{\varphi_{z}}{n}\cdot (\varphi_{z}+\varphi_{x}\cdot \text{sin}\frac{\varphi_{y}}{n})\right)\\ F=\varphi_{y}^{2}+\varphi_{z}^{2}\\ G=\varphi_{z}\cdot \text{cos}\frac{\varphi_{z}}{n}\cdot (\varphi_{z}\cdot \text{cos}\frac{\varphi_{x}}{n}-\varphi_{y}\cdot \text{sin}\frac{\varphi_{x}}{n})\\ H=\varphi_{y}\cdot \text{cos}\frac{\varphi_{y}}{n}\cdot (\varphi_{y}\cdot \text{cos}\frac{\varphi_{x}}{n}+\varphi_{z}\cdot \text{sin}\frac{\varphi_{x}}{n})\\ I=\varphi_{z}\cdot \text{sin}\frac{\varphi_{y}}{n}\cdot \text{sin}\frac{\varphi_{z}}{n}\cdot (\varphi_{y}\cdot \text{cos}\frac{\varphi_{x}}{n}+\varphi_{z}\cdot \text{sin}\frac{\varphi_{x}}{n}) \end{array}$$

Using Mathematica [22], we obtain the following result:

(A.23) $$\varphi =\sqrt{\varphi_{x}^{2}+\varphi_{y}^{2}+\varphi_{z}^{2}}$$
